# The Bloch point in uniaxial ferromagnets as a quantum mechanical object

**DOI:** 10.1186/1556-276X-9-132

**Published:** 2014-03-19

**Authors:** Andriy Borisovich Shevchenko, Maksym Yurjevich Barabash

**Affiliations:** 1 G.V. Kurdymov Institute of Metal Physics, National Academy of Science of Ukraine, 36 Vernadskogo pr., Kyiv 142, 03680, Ukraine; 2 Technical Centre, National Academy of Science of Ukraine, 13 Pokrovskya St, Kyiv 04070, Ukraine

**Keywords:** Quantum tunneling, The bloch point, Domain walls, Vertical bloch lines, uniaxial magnetic film, Quantum depinning, Magnetic field, Potential barrier, Ferromagnetic materials

## Abstract

Quantum effects such as tunneling through pinning barrier of the Bloch Point and over-barrier reflection from the defect potential of one have been investigated in ferromagnets with uniaxial strong magnetic anisotropy. It is found that these phenomena can be appeared only in subhelium temperature range.

## Background

Mesoscopic magnetic systems in ferromagnets with a uniaxial magnetic anisotropy are nowadays the subject of considerable attention both theoretically and experimentally. Among these systems are distinguished, especially domain walls (DWs) and elements of its internal structure - vertical Bloch lines (BLs; boundaries between domain wall areas with an antiparallel orientation of magnetization) and Bloch points (BPs; intersection point of two BL parts) [1]. The vertical Bloch lines and BPs are stable nanoformation with characteristic size of approximately 10^2^ nm and considered as an elemental base for magnetoelectronic and solid-state data-storage devices on the magnetic base with high performance (mechanical stability, radiation resistance, non-volatility) [2]. The magnetic structures similar to BLs and BPs are also present in nanostripes and cylindrical nanowires [3-6], which are perspective materials for spintronics.

It is necessary to note that mathematically, the DW and its structural elements are described as solitons, which have topological features. One of such features is a topological charge which characterized a direction of magnetization vector $\vec{M}$ reversal in the center of magnetic structure. Due to its origin, the topological charges of the DW, BL, and BP are degenerated. Meanwhile, in the low temperature range (*T* < 1 K), $\vec{M}$ vector reversal direction degeneration can be lifted by a subbarier quantum tunneling. Quantum magnetic fluctuations of such type in DWs of various ferro- and antiferromagnetic materials were considered in [7-11]. The quantum tunneling between states with different topological charges of BLs in an ultrathin magnetic film has been investigated in [12].

Note that in the subhelium temperature range, the DWs and BLs are mechanically quantum tunneling through the pining barriers formed by defects. Such a problem for the case of DW and BL in a uniaxial magnetic film with strong magnetic anisotropy has been investigated in [13] and [14], respectively. Quantum depinning of the DW in a weak ferromagnet was investigated in article [15]. At the same time, the BPs related to the nucleation [16-18] definitely indicates the presence of quantum properties in this element of the DW internal structure, too. The investigation of the abovementioned problem for the BP in the DW of ferromagnets with material quality factor (the ratio between the magnetic anisotropy energy and magnetostatic one) *Q* > > 1 is the aim of the present work. We shall study quantum tunneling of the BP through defect and over-barrier reflection of the BP from the defect potential. The conditions for realization of these effects will be established, too.

## Methods

### Quantum tunneling of the Bloch point

Let us consider a domain wall containing vertical BL and BP, separating the BL into two parts with different signs of the topological charge. Introducing a Cartesian coordinate system with the origin at the center of BP, the axis OZ is directed along the anisotropy axis, OY is normal to the plane of the DW. According to the Slonczevski equations [1], one can show that in the region of the domain wall Δ < *r* ≤ Λ, where Δ is the DW width, $r=\sqrt{x^{2}+z^{2}}$ , $\Lambda =\Delta \sqrt{Q}$ is the characteristic size of BL, the Bloch point deforms a magnetic structure of BL, as is described by the following ‘vortex solutions’ [19].

(1) tgϕ=z/x

where *ϕ* = arc*tg M*_*y*_/*M*_*x*_ are the components of the vector $\vec{M}$. In this case, a distribution of the magnetization along the axis OY has the Bloch form: sin*θ* = *ch*^−1^(*y*/Δ), where *θ* is the polar angle in the chosen coordinate system.

It is noted that it is the area which mainly contributes to *m*_BP_ = Δ/*γ*^2^ (*γ* is the gyromagnetic ratio) - the effective mass of BP [19]. It is natural to assume that the abovementioned region of the DW is an actual area of BP.

Taking into account Equation 1 and assuming that the motion of BP along the DW is an automodel form *ϕ* = *ϕ*(*z* − *z*_0_, *x*), *z*_0_ is the coordinate of the BP's center), we can write after a series of transformations the energy of interaction of the Bloch point *W*_*H*_ with the external magnetic field ${\vec{H}}_{y}=-H{\vec{e}}_{y}$ as follows:

(2) $$W_{H}=-M_{S}\pi \Delta z_{0}H\int_{\Delta <\;r\;\leq \Lambda }\text{dxdz}{\left(\left(\cos\phi \frac{\partial \phi }{\partial z}\right)\right|}_{z_{0}=0}\approx -M_{S}\pi^{2}\Delta \Lambda z_{0}H,$$

where *M*_*S*_ is the saturation magnetization.

To describe the BP dynamics caused by magnetic field *H* and effective field of defect *H*_*d*_, we will use the Lagrangian formalism. In this case, using Equation 2 and the ‘potential energy’ in the Lagrangian function $L=m_{\text{BP}}\frac{{\dot{z}}^{2}}{2}\;-\;W\left(z_{0}\right)$, we can write it in such form

(3) $$W\left(z_{0}\right)=-M_{S}^{2}\pi^{2}\Lambda \Delta \int_{0}^{z_{0}}dz^{\prime }\left(H-H_{d}\left(z^{\prime }\right)\right)$$

Expanding *H*_*d*_(*z*_0_) in series in the vicinity of the defect position, its field can be presented in the following form:

(4) $$H_{d}\left(z_{0}\right)=H_{c}\left(1-{\left(z_{0}-d\right)}^{2}/2D^{2}\right)$$

where *H*_*c*_ is the coercive force of a defect, *d* is the coordinate of its center, $D^{-2}={\left(\frac{1}{H_{c}}\frac{\partial^{2}H_{d}}{\partial z_{0}^{2}}\right|}_{z_{0}=d}$, *D* is the barrier width.

It is reasonable to assume that the typical change of defect field is determined by a dimensional factor of given inhomogeneity. It is clear that in our case, $\partial^{2}H_{d}/\partial z_{0}^{2}\sim H_{c}/\Lambda^{2}$ and hence *D* ~ Λ. Note also that the abovementioned point of view about defect field correlates with the results of work [20], which indicate the dependence of coercive force of a defect on the characteristic size of the DW, vertical BL, or BP.

**Figure 1:**
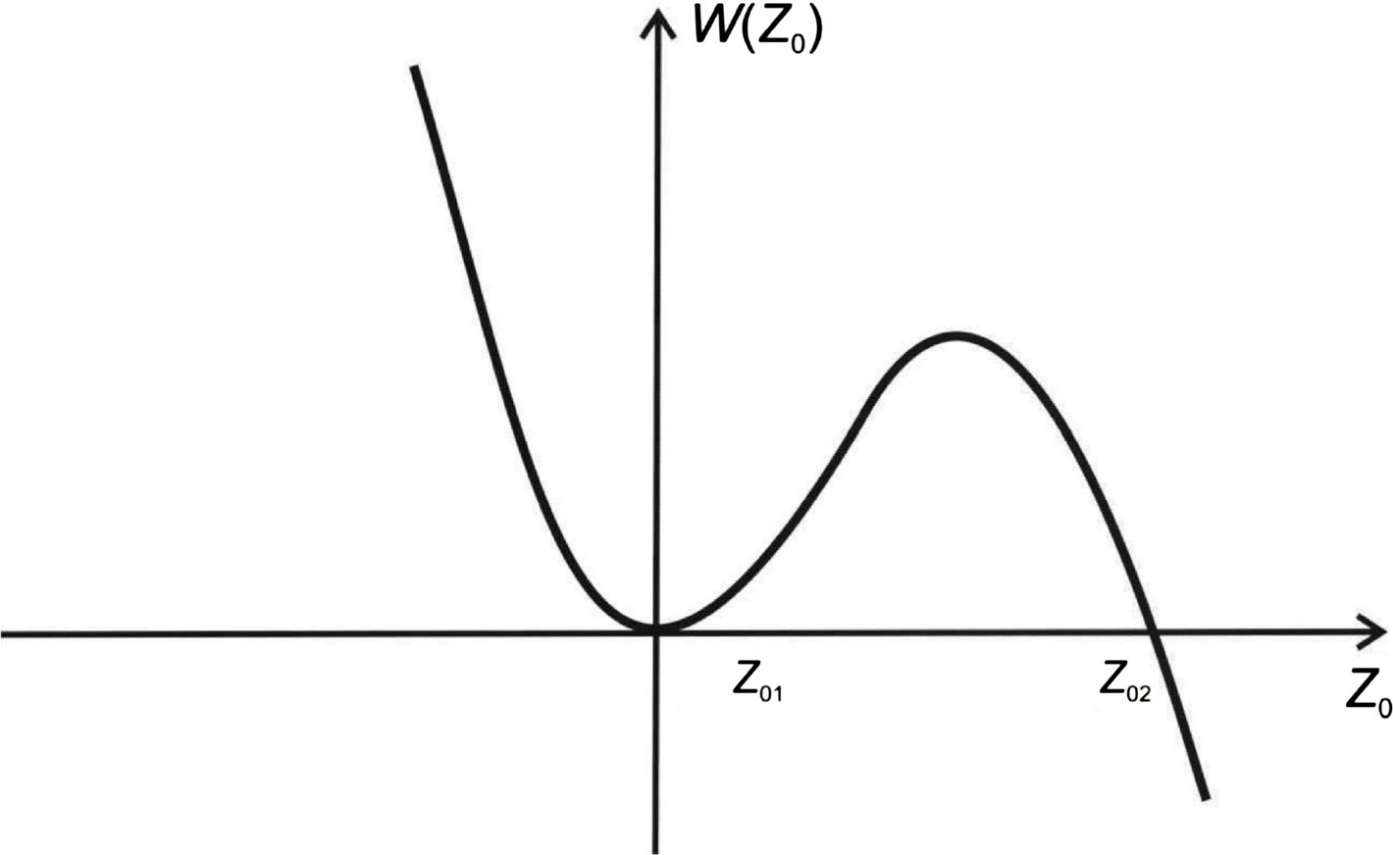
**Potential **$W\left(z_{0}\right)$**caused by magnetic field *H *** **and effective field of defects *H***_**d**_**.**

Substituting Equation 4 into Equation 3, and taking into account that in the point *z*_0_ = 0, the ‘potential energy’ *W* has a local metastable minimum (see Figure 1), we obtain the following expression:

(5) $$W\left(z_{0}\right)=\frac{\pi^{2}Q^{-1/2}M_{S}H_{c}}{2}\left(-\frac{z_{0}^{3}}{3}\;+\;dz_{0}^{2}\right)$$

where $d=\Lambda \sqrt{2\epsilon }$, *ϵ* = 1 − *H*/*H*_*c*_ < < 1 (we are considering the magnetic field values *H* close *H*_*c*_, that decreases significantly the height of the potential barrier). In addition, potential *W*(*z*_0_) satisfies the normalization condition

$$W\left(z_{0,1},z_{0,2}\right)=0$$

where *z*_0,1_ = 0 and $z_{0,2}=3\Lambda \sqrt{2\epsilon }$ are the barrier coordinates.

It should be mentioned that Equation 5 corresponds to the model potential proposed in articles [13-15] for the investigation of a tunneling of DW and vertical BL through the defect.

Following further the general concepts of the Wentzel-Kramers-Brilloin (WKB) method, we define the tunneling amplitude *P* of the Bloch point by the formula

$$P\sim \exp\left(-B\right)$$

where $B=\frac{2}{\hbar }\int_{z_{0,1}}^{z_{0,2}}\left|\dot{z}m_{\text{BP}}\right|\mathrm{dz}$ and *ℏ* is the Planck constant.

After variation of the Lagrangian function L and integration of the obtained differential equation with the boundary condition in the point *z*_0_ = 0, ${\dot{z}}_{0}\to 0,$ and *t* → −*∞*, which corresponds to the pinning of the BP on a defect in the absence of the external magnetic field, we will find the momentum of the BP and, hence, the tunneling exponent

(6) $$B=\frac{2}{\hbar }\int_{z_{0,1}}^{z_{0,2}}\sqrt{2m_{\text{BP}}W\left(z\right)}\mathrm{dz}$$

Taking into account Equation 5, the expression (6) can be rewritten in the following form:

(7) $$B=\frac{8\Delta^{3}Qh_{c}^{1/2}\epsilon^{5/4}}{\hbar \omega_{M}}{\left(4\pi M_{S}\right)}^{2}$$

where *h*_*c*_ = *H*_*c*_/8*M*_*S*_, *ω*_*M*_ = 4*πγM*_*S*_.

Temperature *T*_*c*_ at which the quantum regime of the BP motion takes place can be derived from relations (5) and (7), taking into account the relation Tc=Wmax/kBB, where *W*_max_ is the maximal value of the potential barrier, *k*_*B*_ is the Boltzmann constant. Thus, in accordance with the above arguments, we obtain

$$W_{\text{max}}=2\frac{\sqrt{2}}{3}Q{\left(4\pi M_{S}\right)}^{2}\Delta^{3}h_{c}\epsilon^{3/2}$$

and

(8) $$T_{c}=\frac{\sqrt{2}\epsilon^{1/4}h_{c}^{1/2}\hbar \omega_{M}}{12k_{B}}$$

Substituting into the expressions (7) and (8), the numerical parameters corresponding to uniaxial ferromagnets: *Q* ~ 5–10, Δ ~ 10^−6^ cm, 4*πM*_*S*_ ~ (10^2^ − 10^3^) Gs, *H*_*c*_ ~ (10 − 10^2^) Oe [19] (see also articles [20,21], in which the dynamic properties of BP in yttrium-iron garnet were investigated), *γ* ~ 10^7^ Oe^−1^ s^−1^, for *ϵ* ~ 10^−4^ − 10^−2^, we obtain *B* ≈ 1–30 and *T*_*c*_ ~ (10^−3^ − 10^−2^) К.

The value obtained by our estimate *B* ≤ 30 agrees with corresponding values of the tunneling exponent for magnetic nanostructures [22], which indicate the possibility of realization of this quantum effect. In this case, as can be seen from the determination of the BP effective mass, in contrast to the tunneling of the DW and vertical BL through a defect, the process of the BP tunneling is performed via the ‘transfer’ of its total effective mass through the potential barrier.

Following the integration of the motion equation of the BP obtained via the Lagrangian function variation, we find the its instanton trajectory *z*_in_ and the instanton frequency of the Bloch point *ω*_in_ (see review [23]), which characterize its motion within the space with an ‘imaginary’ time *τ* = *it*: from the point *z*_0,1_ = 0 at *τ* = −*∞* to the point $z_{0,2}=3\Lambda \sqrt{2\epsilon }$ at *τ* = 0 and back to the point *z*_0,1_ at *τ* = *∞*

(9) $$z_{\mathrm{in}}=3\Lambda \sqrt{2\epsilon }/ch^{2}\left(\omega_{\mathrm{in}}\tau \right),\omega_{\mathrm{in}}=\omega_{M}h_{c}^{1/2}{\left(2\epsilon \right)}^{1/4}/2$$

Further, in defining the instanton frequency, we shall consider the validity of use of WKB formalism for the description of the BP quantum tunneling. As known [24], the condition of applicability of the WKB method is the fulfillment of the following inequality:

(10) $$\mathrm{m\hbar}\left|F\right|/p^{3}<<1$$

where *p* is momentum, *m* is the quasiparticle mass, and *F* is the force acting on it.

In our case $F=m_{\text{BP}}\omega_{\mathrm{in}}^{2}\xi$, *p* = *m*_BP_*ω*_in_*ξ*, $\xi \sim \Lambda \sqrt{2\epsilon }$. Then, taking into account Equation 9, we will rewrite Equation 10 in the following way:

(11) $$\hbar \gamma^{2}\omega_{M}^{-1}h_{c}^{-1/2}{\left(2\epsilon \right)}^{-5/4}Q^{-1}/\Delta^{3}<<1$$

Setting the abovementioned parameters of the ferromagnets and defect into Equation 11, it is easy to verify that this relationship is satisfied, that in turn indicates the appropriateness of use of the WKB approximation in the problem under consideration.

Let us estimate the effect of dissipation on the tunneling process of the BP. To do this, we compare the force *F*, acting on the quasiparticle, with the braking force $\tilde{F}$,which in our case is approximately $\alpha \omega_{M}\omega_{\mathrm{in}}\Lambda \sqrt{2\epsilon }m_{\text{BP}}$, where *α* ~ 10^−3^ − 10^−2^ is the magnetization decay parameter. Taking into account the explicit form of *F*, we obtain

$$\tilde{F}/F=2\alpha /h_{c}^{1/2}{\left(2\epsilon \right)}^{1/4}$$

The analysis of this expression shows that $\tilde{F}/F<<1$ at 10^−2^ ≤ *h*_*c*_ ≤ 10^−1^, *ϵ* ~ 10^−4^ − 10^−2^ and *α* ~ 10^−3^ − 10^−2^. The obtained result indicates that at the consideration of the BP quantum tunneling process, the effect of breaking force can be neglected.

Note also that the mechanism of breaking force has been investigated in the work [25] and is associated with the inclusion of relaxation terms of exchange origin in the Landau-Lifshiz equation for magnetization of a ferromagnet [26].

## Results and discussion

### The over-barrier reflection of the Bloch point

In the above, it was mentioned that tunneling of DW and vertical BL is carried out via sub-barrier transition of small parts of the area of DW or the length in case BL. In this case, both DW and vertical BL are located in front of a potential barrier at a metastable minimum that makes possible the process of their tunneling. At the same time, the BP depinning occurs via ‘transmission’ through the potential barrier instantly of entire effective mass of the quasiparticle. This result indicates that the presence of a metastable minimum in the interaction potential of BP with a defect (in contrast to DW or BL) is not necessary. Moreover, it means that there exists a possibility of realization for BP of such quantum effect as over-barrier reflection of a quasiparticle from the defect potential. In this case, the velocity at which BP ‘falls’ on the barrier may be determined by a pulse of magnetic field applied to the BP. And, as we shall see bellow, the potential of interaction between the BP and a defect has a rather simple form. Obviously, the effect is more noticeable in the case when the BP energy is not much greater than the height of the potential barrier *U*_0_.

Using the formula (2), we represent the dynamics equation for the BP in a pulsed magnetic field *H*_*y*_(*t*) = *H*_0_*χ*(1 − *t*/*T*) in the form

(12) $$m_{\text{BP}}\partial v/\partial t\;+\;\tilde{F}=\pi^{2}\Lambda \Delta M_{S}H_{y}\left(t\right)$$

where *v* = ∂*z*_0_/∂*t* is the BP velocity, *χ*(1 − *t*/*T*) is the Heaviside function, *H*_0_ is the amplitude, and *T* is the pulse duration.

By integrating the Equation 12 for $T\leq t<<\alpha^{-1}\omega_{M}^{-1}$, we find the velocity of the Bloch point at the end of the magnetic field pulse: *v*(*t*) = *π*^2^*M*_*S*_ΛΔ*H*_0_*T*/*m*_BP_. Accordingly, the energy of the BP in current time range *E*_BP_ is given by

(13) $$E_{\text{BP}}=m_{\text{BP}}v^{2}/2=\pi^{2}\omega_{M}^{2}T^{2}\Lambda^{2}\Delta H_{0}^{2}/32$$

Note that the study, performed for time $t<<\alpha^{-1}\omega_{M}^{-1}$ (or with taking into account the value of the magnetization decay *ω*_*M*_*t* < < 10^2^ − 10^3^), allows us to neglect the effect on the process of the braking force $\tilde{F}\sim \alpha \omega_{M}m_{\text{BP}}v.$

We assume that defect is located at *z*_0_ = 0. Then, by expanding the potential of interaction of BP with the defect, *U*_*d*_(*z*_0_), in a series near this point and taking Equation 2 into account, we can write down

(14) $$U_{d}\left(z_{0}\right)=U_{0}\left(1-z_{0}^{2}/2\Lambda^{2}\right)$$

where in accordance with the formula (2), the height of the potential barrier is *U*_0_ = *π*^2^Λ^2^Δ*M*_*S*_*H*_*c*_.

Note that phenomenological expression for defect-effective field *H*_*d*_ (see formula (4)) follows from the series expansion of the potential *U*_*d*_(*z*_0_) near the inflection point. It was at this point that there is maximum field of defect. It is natural to assume that if BP has overcome the barrier in this point, then the tunneling process is probable in general.

Using the WKB approximation, and following the formalism described in [27,28], we determine the coefficient of over-barrier reflection of the Bloch Point *R* by the formula

(15) $$R=e^{-\beta }$$

where β=−2ℏIm∫z0,1∗z0,2∗dz2mBPEBP−Udz$z_{0,2}^{*}$, and $z_{0,2}^{*}$ are the roots of the equation *E*_BP_ − *U*_*d*_(*z*_0_) = 0.

Taking into account the expression for the potential (14), from Equation 15, we find

(16) $$\beta =\pi \sqrt{2m_{\text{BP}}}E_{\text{BP}}\Delta \epsilon^{\prime }/\hbar \sqrt{U_{0}}$$

where the parameter *ϵ*′ = (*E*_BP_ − *U*_0_)/*E*_BP_ < < 1 (recall that we consider the case when the energy *E*_BP_ close to *U*_0_).

Using the formula (13), Equation 16 can be rewritten as

(17) $$\beta =\pi {\left(2M_{S}H_{c}\right)}^{1/2}\epsilon^{\prime }\gamma^{-1}\Delta^{3}Q^{1/2}/\hbar$$

Substituting into the expressions (15) and (17), the ferromagnet and defect parameters, at *ϵ*′ ≥ 5 × 10^−5^ we obtain *R* ≤ 0.1, which is in accordance with criterion of applicability of Equation 15 (see [28]).

Note that from Equations 15 and 16, it follows that *R* → 0 at *U*_0_ → 0, i.e., we obtain a physically consistent conclusion about the disappearance of the effect of over-barrier reflection in the absence of a potential barrier.

Based on the obvious relation, $\tau \sim \Delta {\left(\frac{m_{\text{BP}}}{U_{0}}\right)}^{1/2}=\frac{4}{\omega_{M}}{\left(\frac{M_{S}}{H_{c}}\right)}^{1/2}Q^{-1/2}$ and the numerical data, given above, we determine *τ*, the characteristic time of interaction of BP with the defect 0.6 ≤ *ω*_*M*_*τ* ≤ 2.3. It is easy to see that *τ* satisfies the relation *ω*_*M*_*τ* < *ω*_*M*_*t* ~ 10 − 10^2^, which together with an estimate for *R* indicates on the possibility of the quantum phenomenon under study. In this case, the analysis of expressions (13) and (14) shows that the amplitude of a pulsed magnetic field is *H*_0_ ~ 4*π*(*M*_*S*_*H*_*c*_)^1/2^/*ω*_*M*_*T* < 8*M*_*S*_, which is consistent with the requirement for values of the planar magnetic fields applied to DW in ferromagnets [1].

Let us consider the question about validity of applicability of the WKB approximation to the problem under consideration. Since in the given case *E*_BP_ ≈ *U*_0_, then the conditions of ‘quasi-classical’ behavior of the Bloch point and the potential barrier actually coincide and, in accordance with [24], are reduced to the fulfillment of the inequality

(18) $$\delta z_{0}\sqrt{m_{\text{BP}}U_{0}}/\hbar >>1$$

where $\delta z_{0}=\Delta \sqrt{2\left(E_{\text{BP}}-U_{0}\right)/U_{0}}\approx \Delta \sqrt{2\epsilon^{\prime }}.$

Using the explicit form of *U*_0_, Equation 18 can be rewritten as

$$\pi \gamma^{-1}\Delta^{3}{\left(M_{S}H_{c}\right)}^{1/2}Q^{1/2}{\left(\epsilon^{\prime }\right)}^{1/2}>>\hbar$$

An analysis of this inequality shows its fulfillment for the values *ϵ*′ ≥ 10^−4^, that in fact is a ‘lower estimate’ for this parameter. In a critical temperature $T_{c}^{*}$, corresponding to the given effect, we determine from the exponent in the formula (15) using the relation $k_{B}T_{c}^{*}=\beta^{-1}\left(E_{\text{BP}}-U_{0}\right)$. Then, taking into account Equation 17, finally, we get

(19) $$T_{c}^{*}=\frac{\hbar U_{0}^{1/2}}{\pi k_{B}\sqrt{2m_{\text{BP}}}\Delta }=\frac{\hbar \gamma }{\sqrt{2}k_{B}}{\left(M_{S}H_{c}\right)}^{1/2}$$

An estimate of the expression (19) shows that $T_{c}^{*}\sim \left(10^{-3}-10^{-2}\right)$ K. Such values of $T_{c}^{*}$ are in the same range with critical temperatures for processes of quantum tunneling of DW [13], vertical BL [14] and BP through a defect. This fact indicates the importance of considering the effect of over-barrier reflection of BP in the study of quantum properties of these magnetic inhomogeneities.

## Conclusions

It is shown that in the subhelium temperature range, the Bloch point manifest themselves as a quantum mechanical object. Thus, the BP may tunnel through the pining barrier formed by the defect and over-barrier reflection from the defect potential. In this case, since the over-barrier reflection of the BP and sub-barrier tunneling of the BP occur in pulse and permanent magnetic fields, respectively, the practical possibility to study these quantum phenomena separately exists. Moreover, the experimental realization of the mentioned phenomena can be the basis for the creation of new methods of diagnostic of ferromagnetic materials and sensitive methods for studying an internal structure of their DWs.

## Competing interests

The authors declare that they have no competing interests.

## Authors’ contributions

ABS and MYB read and approved the final manuscript.
